# Interruption of ghrelin signaling in the PVN increases high-fat diet intake and body weight in stressed and non-stressed C57BL6J male mice

**DOI:** 10.3389/fnins.2013.00167

**Published:** 2013-09-17

**Authors:** Zachary R. Patterson, Tamara Parno, Albert M. Isaacs, Alfonso Abizaid

**Affiliations:** Department of Neuroscience, Carleton UniversityOttawa, ON, Canada

**Keywords:** ghrelin, PVN, food intake, body weight, hypothalamus, stress, social defeat

## Abstract

Chronic social stress has been associated with increased caloric intake and adiposity. These effects have been linked to stress induced changes in the secretion of ghrelin, a hormone that targets a number of brain regions to increase food intake and energy expenditure and promote increased body fat content. One of the brain sites targeted by ghrelin is the hypothalamic paraventricular nucleus (PVN), a region critical for both the regulation of the stress response and the regulation of energy balance. Given these data, we examined the contribution of ghrelin receptors in the PVN to the metabolic and behavioral changes that are seen during chronic social stress in mice. To do this, mice were implanted with cannulae attached to osmotic minipumps and delivering either vehicle or the ghrelin receptor (growth hormone secretagogue receptor) antagonist [D-Lys-3]-GHRP-6 (20 nmol/day/mouse). Following a week of recovery, half of the animals in each group were exposed to chronic social defeat stress for a period of 3 weeks whereas the other half were left undisturbed. During this time, all animals were given *ad libitum* access to standard laboratory chow and presented a high-fat diet for 4 h during the day. Results showed that the ghrelin receptor antagonism did not decrease stressed induced caloric intake, but paradoxically increased the intake of the high fat diet. This would suggest that ghrelin acts on the PVN to promote the intake of carbohydrate rich diets while decreasing fat intake and blockade of ghrelin receptors in the PVN leads to more consumption of foods that are high in fat.

## Introduction

In mammals, environmental stressors result in the activation of a number of homeostatic and allostatic physiological mechanisms that enable the organism to deal with the impending threat (McEwen, 2007). One of these mechanisms involves the activation of the hypothalamic pituitary adrenal (HPA) axis, a major neuroendocrine system that controls the response to stress. Thus, in the face of a stressor, parvocellular cells in the paraventricular nucleus (PVN) of the hypothalamus secrete corticotropin releasing hormone (CRH) onto the portal system in the median eminence and onto the anterior pituitary. Here CRH acts on corticotropes to elicit the secretion of adrenal corticotropin releasing hormone (ACTH) into the blood stream (Smith and Vale, 2006). Increased circulating levels of ACTH stimulate cells in the adrenal cortex to release glucocorticoids (Stratakis and Chrousos, 1995; Smith and Vale, 2006). Increased levels of glucocorticoids are important for a number of reasons that include the release of glucose and glycogen stores from the liver and muscle, and the release of insulin from the pancreas facilitating the generation of energy required to meet the energetic demands posed by the stressor (Black, 2006). Glucocorticoids then provide a negative feedback signal to the hypothalamus and hippocampus to terminate any further activation of the HPA axis (McEwen, 2007).

Recently, the orexigenic hormone ghrelin has emerged as another hormone that is secreted in response to stressors and one that may produce some of the metabolic changes required to deal with the energetic demands posed by the stressors (Patterson et al., 2013). In addition to increasing food intake and growth hormone secretion, ghrelin promotes the utilization of carbohydrates as a fuel source, while preventing the use of lipids, resulting in increases in body weight due to increases in body fat (Kojima et al., 1999; Tschop et al., 2000). Moreover, these effects are produced by ghrelin acting centrally (Tschop et al., 2000; Nakazato et al., 2001; Patterson et al., 2013). Under normal conditions, ghrelin is secreted in circadian patterns, or in anticipation of scheduled meals (Cummings et al., 2001; Drazen et al., 2006). Nevertheless, ghrelin is also secreted in response to acute stressors, and circulating ghrelin levels are higher in chronically stressed animals (Lutter et al., 2008; Patterson et al., 2009, 2013). Thus, animals that are exposed to repeated social stressors often show increased caloric intake, body weight, and adiposity in tandem with increased ghrelin concentrations (Lutter et al., 2008; Bartolomucci et al., 2009; Kumar et al., 2013; Patterson et al., 2013). More importantly, ghrelin mediates these effects through its action on brain sites that contain the GHSR (Patterson et al., 2013). These data support the notion that stress generates a metabolic challenge, and that ghrelin is released as part of the physiological mechanisms that are generated to meet this challenge. Interestingly, mice with mutations to the only known ghrelin receptor, the growth hormone secretagogue receptor (GHSR), are more susceptible to develop depressive like behaviors after chronic social stress compared to their wild type (WT) littermates (Lutter et al., 2008).

While it is clear that stress-induced ghrelin secretion affects food intake and energy balance through central actions (Patterson et al., 2013), not much is known about the relative contribution of different ghrelin-sensitive brain regions, many of which are implicated in the behavioral and metabolic responses to stress. Among these regions, the PVN stands out given its role in the regulation of the HPA axis as well as in the modulation of sympathetic responses, and its connections with peripheral tissues such as the liver, pancreas and adipose tissues (Hill, 2013). Ghrelin receptors are found within the PVN although it appears that they do not co-localize with CRH producing neurons (Guan et al., 1997; Zigman et al., 2006; Cabral et al., 2012). Nevertheless, ghrelin acting on other cells in the PVN could be responsible for some of the behavioral and metabolic alterations that are produced by exposure to chronic social stress (Cabral et al., 2012). To examine this possibility, we exposed mice to chronic social defeat stress for a period of 21 days while being infused with a ghrelin receptor antagonist ([D-Lys-3]-GHRP-6) or vehicle (0.9% saline) and compared their metabolic phenotypes with non-stressed controls receiving the same drug or vehicle infusions.

## Materials and methods

### Animals

Male C57BL6J mice weighing 20–22 g were obtained from Charles River farms, St. Constant, Quebec as experimental subjects. Mice (*N* = 48) were housed under standard laboratory conditions with *ad libitum* access to mouse chow (3.3 kcal/g, with 70% of calories derived from carbohydrates) and tap water in addition to daily 4-h high fat diet containing 60% caloric content from fat (TD 06414, Harlan). The calculated metabolized energy of the high fat diet was 5.1 kcal/g with 60.0% calories from fat. Male CD-1 retired breeder mice weighing 40–50 g, also obtained from Charles River Farms, were used as aggressors in the chronic social defeat stress paradigm. Food intake (both standard laboratory chow and high-fat diet) and body weight were weighed and recorded daily by the experimenters at 9:00 AM. The high-fat diet was available from 10:00 AM to 2:00 PM daily. Following the baseline period, mice were separated into 4 experimental groups as follows: vehicle stressed (*n* = 12), vehicle non-stressed (*n* = 12), [D-Lys-3]-GHRP-6 non-stressed (*n* = 12) and [D-Lys-3]-GHRP-6 stressed (*n* = 12). Due to attrition of animals throughout the 28-day cannula-minipump infusion process, as well as misplaced cannulae, the final group numbers used for data analysis were as follows: vehicle stressed (*n* = 6), vehicle non-stressed (*n* = 10), [D-Lys-3]-GHRP-6 non-stressed (*n* = 4) and [D-Lys-3]-GHRP-6 stressed (*n* = 6). All procedures documented were approved by the Carleton University Animal Care Committee and the guidelines of the Canadian Council on Animal Care were followed.

### Stereotaxic surgery—chronic delivery of ghrelin receptor antagonist into the paraventricular nucleus of the hypothalamus

Prior to surgery, we conducted a 2-week baseline where measures of food intake (both standard laboratory chow and high fat diet) and body weight were recorded daily. After baseline, mice were anesthetized using isofluorane mixed with oxygen (4%) and implanted with an intracranial cannula connected to an osmotic minipump delivering either saline or the ghrelin receptor antagonist [D-Lys-3]-GHRP-6. To do this, the mouse's head was shaved and secured onto a mouse stereotaxic apparatus while anaesthetized (Kopf Instruments, Tujunga, CA). The scalp was cleaned with surgiprep and privodine to provide an aseptic canvas. Tear gel was applied to prevent dehydration of the eyes. A midline incision was made and the skin was retracted for a clear visualization of bregma. A 28 gauge stainless steel unilateral cannula (Alzet Brain Infusion Kit; Order #004760) coupled to an osmotic mini-pump (Alzet Mini-Osmotic Pump—Model 1004; flow rate: 0.11 μL/h for 28 days) using a polyethylene catheter and was implanted into the PVN of the hypothalamus. Stereotactic coordinates of the cannula, relative to bregma, were AP −0.94 mm, ML −1.75 mm and DV 4.83 mm (Paxinos and Franklin, 1997). Mini-pumps were filled with 100 μL of sterile saline (0.9% NaCl) to the control group while the experimental group had mini-pumps filled with 100 μL of the ghrelin receptor antagonist ([D-Lys-3]-GHRP-6) solution (Peptides International; 20 nmol/day/mouse). The implant was secured with contact and dental cement. When the cement was dry, the dorsal portion of the skin was separated from the muscle using blunt dissection to implant the mini-pump subcutaneously. The incision was closed using silk surgical sutures. Polysporin and Lidocaine were applied topically to the surgical site to prevent bacterial infection and pain, respectively. Mice were also injected with a low dose of meloxicam (Metacam, 0.1 mL of 5 mg/mL) to provide postoperative analgesia. Following surgery, mice were moved to a recovery area in a clean cage with a heating pad. Upon wakening, mice were monitored closely for optimal recovery for a total of 7 days following surgery.

### Chronic social defeat stress paradigm

Following the 1 week recovery period after surgery, half of the saline and half of the ghrelin receptor antagonist infused mice were taken from their room and transported to another room where each of them was housed in a cage inhabited by a much larger sexually experience male CD-1 mouse for the next 21 days (Patterson et al., 2013). The experimental mice were protected from the aggressive CD-1 mice by an acrylic divider and wire mesh, yet olfactory, visual, and auditory contact was maintained with the resident for the entire 21-day stress period. Each day, the divider was removed and the animals were permitted to interact until the experimental mouse was subdued or until 15 min had passed. Measurements of regular chow intake, high fat diet intake, and body weight were recorded each day during the stress period.

### Blood plasma collection

At the end of the 21 days of chronic social defeat stress, mice in the stress group were sacrificed by rapid decapitation, the morning following the last social interaction with the dominant mouse. Non-stressed mice were sacrificed on the same day. Glucose concentrations from trunk blood samples were recorded using Accuchek^®^ (Aviva) glucose meter and strips. Blood samples were also collected for analyses of plasma corticosterone (CORT) content. These samples were analyzed using a radioimmunoassay kit according to the manufacturer's protocol (ICN Biomedicals, CA, USA). Inter assay variability was less than 10%.

### Statistics

All data were analyzed using 2 × 2 ANOVAs with drug ([D-Lys-3]-GHRP-6 vs. 0.9% saline) and treatment (stress vs. non-stress) as the between group factors, unless otherwise stated. Data from animals with misplaced cannula were not included in the analyses. All analyses were preceded with a Levene's Test of Quality of Error Variance to ensure the homogeneity of variance assumption was satisfied. The limit for statistical significance was set at α = 0.05. All mice with misplaced cannula were eliminated from all statistical analysis.

## Results

### Histology

**Figure 1:**
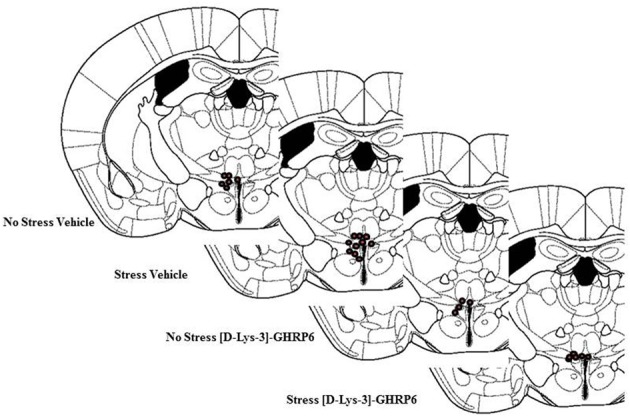
**Location of cannulae placement in animals from each group**. Animals with missed cannulae placements were not included in this figure or in the analyses.

Postmortem analyses of cannulae placements showed that six mice had misplaced cannulae (Vehicle non-stressed miss, *n* = 1; Vehicle stressed miss, *n* = 1; [D-Lys-3]-GHRP-6 non-stressed miss, *n* = 2; [D-Lys-3]-GHRP-6 stressed miss, *n* = 2). The location of the cannulae for animals in each group is shown in Figure 1. As seen in this figure, most placements were located in the dorsolateral portion of the PVN. Because the cannulae were inserted on an angle (18°), most missed cannulae were placed too far lateral to the PVN, with only a few placed too far dorsal. The data from these animals were not included in the analyses given that there were only one or two animals from each group that had misplaced cannulae. Nevertheless, none of the animals with misplaced cannulae and receiving [D-Lys-3]-GHRP-6 showed the effects observed below suggesting that these effects were caused by localized action of the drug onto the PVN and not at sites outside of this region.

### Stress increases caloric intake in mice and this effect is not attenuated by GHSR antagonism in the PVN

**Figure 2:**
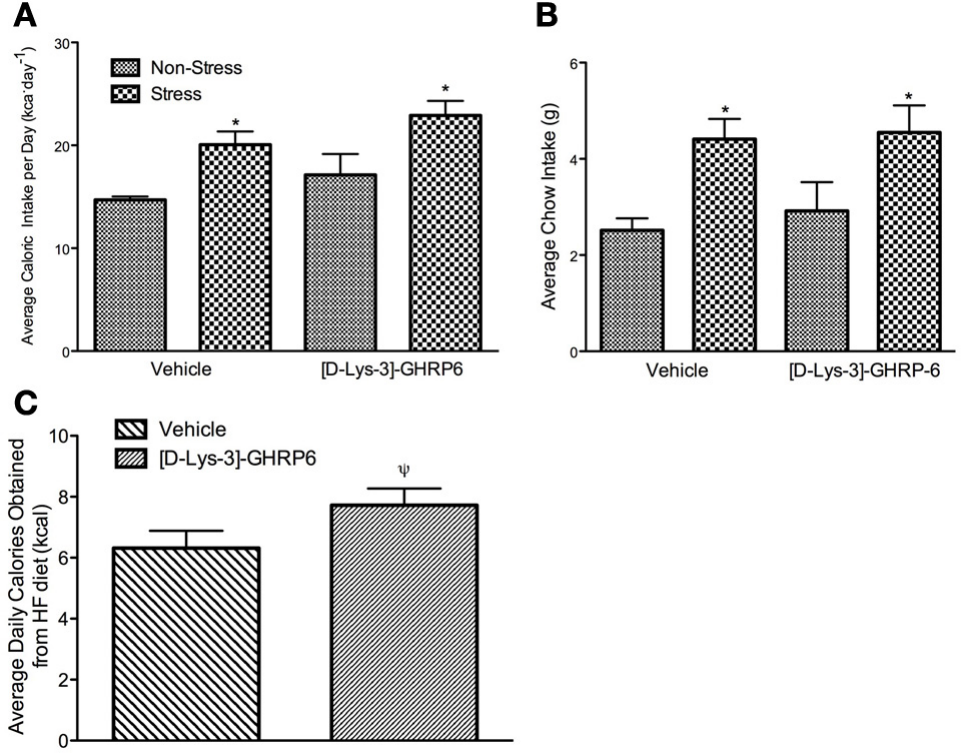
**(A)** Average daily caloric intake (calculated as the sum of calories consumed from standard laboratory chow and high-fat diet), **(B)** average daily standard laboratory chow intake, **(C)** average number of calories consumed from the high fat diet per day for animals receiving either vehicle (0.9% saline) or 20 nmol/day of [D-Lys3-]-GHRP-6; stressed and non-stressed animals are collapse to illustrate main effect of drug. All values are expressed as mean ± s.e.m. ^*^p < 0.05 relative to non-stressed controls. ^ψ^*p* = 0.05 relative to vehicle treated animals.

Total caloric intake was calculated daily throughout the study by adding the caloric content of standard laboratory chow and high-fat diet. At the end of the baseline period, mice were assigned to each of the experimental groups after being matched for total caloric intake. As such, there were no group differences in the amount of calories consumed during the baseline (data not shown). During the week that followed the surgical implantation of the cannulae, mice continued to eat similar amounts of calories and there were no differences between any of the groups. During the stress period, there was no significant interaction between drug and treatment on caloric consumption [interaction, *F*_(1, 22)_ = 0.019, *p* > 0.05], nor was there an effect of [D-Lys-3] GHRP-6 treatment [main effect of drug, *F*_(1, 22)_ = 3.099, *p* > 0.05]. There was, however, a significant treatment effect where stressed mice increased their total daily caloric intake, and this effect was not attenuated by GHSR antagonism [main effect of stress, *F*_(1, 22)_ = 11.77, *p* < 0.05; See Figure 2A].

The increase in caloric intake seen in stressed animals was due primarily to an increase in the daily intake of regular chow. Both vehicle and [D-Lys-3] GHRP-6 treated mice consumed equivalent amounts of chow before the stress period began (*p* > 0.05). Following the stress period, there was no significant interaction between drug and treatment on the consumption of regular chow [interaction, *F*_(1, 22)_ = 0.063, *p* > 0.05]. However, we did observe a significant treatment effect, where stressed mice rapidly increased their intake of regular laboratory chow regardless of the infusion they received in the PVN [main effect of stress, *F*_(1, 22)_ = 10.48, *p* < 0.05; See Figure 2B].

### Blocking GHSR in the PVN increases the intake of the high fat diet

Given that stress has been associated with increased consumption of diets that are calorically dense and highly palatable, we next examined if chronic social defeat stress would alter the intake of a high-fat diet. Mice in all groups ate little of the high fat diet early in the baseline period but increased their intake steadily over time, so that by the end of the baseline period, they were consuming about 37% ± 4.7 of their daily caloric intake from this diet. There was no significant interaction between treatment and drug on the proportion of calories obtained from the high-fat diet during the stress period [interaction, *F*_(1, 22)_ = 0.411, *p* > 0.05]. However, there was a significant treatment effect wherein the proportion of calories obtained from the high fat diet decreased in all animals that were stressed [main effect of stress, *F*_(1, 22)_ = 4.32, *p* < 0.05; data not shown]. Interestingly, there was also an effect of drug wherein mice receiving [D-Lys-3] GHRP-6 infusions into the PVN increased the total amount of high fat diet consumed, relative to animals receiving vehicle infusions regardless of their treatment conditions [main effect for drug, *F*_(1, 22)_ = 4.25, *p* = 0.051; See Figure 2C].

**Figure 3:**
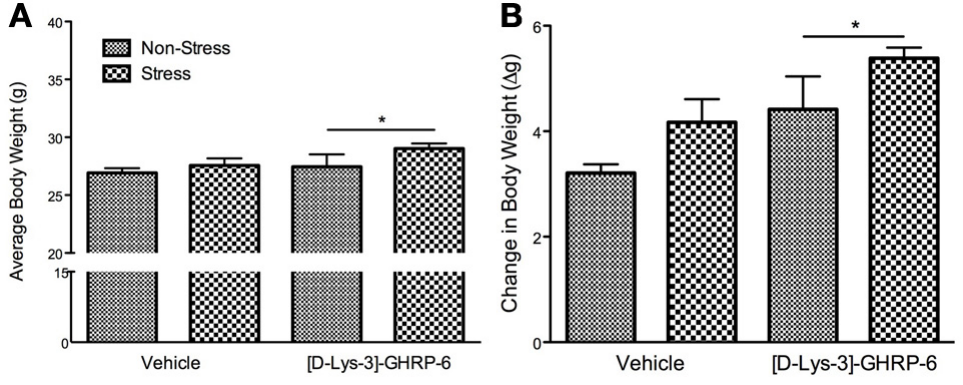
**(A)** Body weight at the end of the 3 week chronic social defeat paradigm, and **(B)** the change of body weight during the stress period. All values are expressed as mean ± s.e.m. ^*^p < 0.05 relative to animals receiving vehicle infusions.

The increase in the total amount of fat consumed by animals infused with [D-Lys-3] GHRP-6 into the PVN was reflected in their body weight at the end of the stress period. There was no significant interaction between drug and treatment on the change in body weight during the stress period [interaction, *F*_(1, 22)_ = 0.00, *p* > 0.05] or on the average body weight at the end of the stress period [interaction, *F*_(1, 22)_ = 0.425, *p* > 0.05]. There was, however, a significant effect of drug infusions on the change in body weight, wherein mice treated chronically with [D-Lys-3] GHRP-6 weighed significantly more at the end of the stress period compared to animals treated with vehicle, regardless of their treatment group [main effect of drug, *F*_(1, 22)_ = 12.49, *p* < 0.05; See Figure 3A].

The effects of [D-Lys-3]-GHRP-6 on body weight were more pronounced when the data were expressed as a change from baseline, and a significant drug effect demonstrates that animals treated with [D-Lys-3]-GHRP-6 gained more weight than those treated with saline during the stress period [main effect for drug, *F*_(1, 22)_ = 7.32, *p* < 0.05; See Figure 3B]. Furthermore, there was a significant main effect of treatment on body weight gain. As expected, stressed mice gained significantly more weight than their non-stressed controls, however, this occurred independently of their drug treatment [main effect of stress, *F*_(1, 22)_ = 4.65, *p* < 0.05; See Figure 3B].

### [D-Lys-3]-GHRP6 infusions into the PVN does not influence stress-induced hyperglycemia but does mediate stress-induced CORT secretion

**Figure 4:**
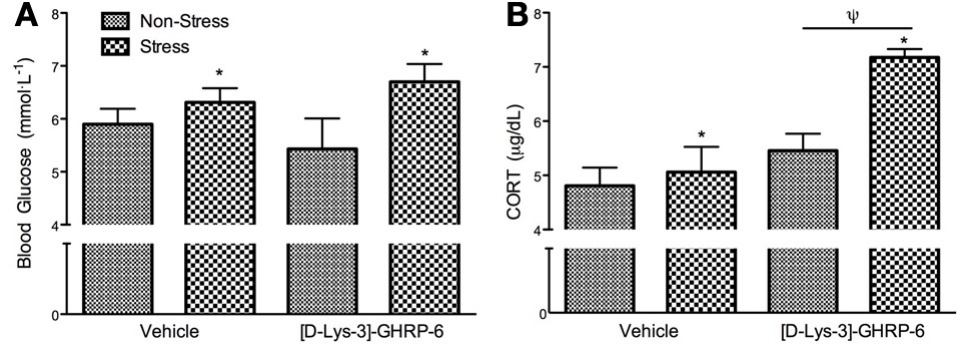
**(A)** Plasma blood glucose levels and **(B)** plasma corticosterone levels in trunk blood collected at the time of sacrifice. All values are expressed as mean ± s.e.m. ^*^p < 0.05 relative to non-stressed animals; ^ψ^*p* < 0.05 relative to vehicle treated animals.

Following sacrifice by rapid decapitation, trunk blood was analyzed for blood glucose and corticosterone. There was no interaction between drug and treatment on circulating blood glucose at the time of sacrifice [interaction, *F*_(1, 22)_ = 1.238, *p* > 0.05]. As expected, mice exposed to chronic social defeat stress had higher basal glucose levels at the time of sacrifice [Figure 4A; main effect of stress, *F*_(1, 22)_ = 5.6, *p* < 0.05], and this effect was not altered by [D-Lys-3] GHRP-6 infusions into the PVN (*p* > 0.05). Similarly, stressed mice had significantly higher levels of circulating CORT at the time of sacrifice compared to non-stressed controls, independent of drug treatment [main effect of stress, *F*_(1, 22)_ = 12.263, *p* < 0.01]. Furthermore, animals receiving [D-Lys-3]-GHRP-6 infusions into the PVN tended to have higher circulating CORT at the time of sacrifice regardless of their treatment group [main effect of drug, *F*_(1, 22)_ = 4.118, *p* = 0.058], and this main effect appeared to come from increased plasma CORT in samples collected from stressed [D-Lys-3]-GHRP-6 infused animals (Figure 4B). There was, however, no interaction effect between drug and treatment on circulating plasma CORT at the time of sacrifice [interaction, *F*_(1, 22)_ = 2.788, *p* = 0.110].

## Discussion

Ghrelin is a peptide that acts centrally to increase food intake and to alter metabolism by promoting the oxidation of carbohydrates while sparing the oxidation of fatty acids as substrate, ultimately increasing adipose tissue accumulation (Tschop et al., 2000). The effects of ghrelin on food intake and carbohydrate utilization have been mimicked by direct infusions of ghrelin onto the PVN (Currie et al., 2005). Similar to chronic delivery of ghrelin, acute and chronic social defeat stress increase the concentrations of the active form of ghrelin in plasma, but also result in higher caloric intake, and in some cases increased body weight, effects that are attenuated in socially defeated ghrelin receptor KO mice (Lutter et al., 2008; Davies et al., 2009; Raspopow et al., 2010; Kumar et al., 2013; Patterson et al., 2013). Interestingly, this type of social stress causes mice to alter their metabolism to promote the utilization of carbohydrates, ultimately leading to increased adipose stores (Davies et al., 2009; Patterson et al., 2013). In contrast, ghrelin receptor KO mice continue to use fat as a substrate for nutrients and tend to lose body fat when chronically stressed, ultimately showing depletion of adipose tissue (Patterson et al., 2013). Furthermore, ghrelin seems to produce these metabolic effects centrally as chronic delivery of [D-Lys-3]-GHRP-6 into the cerebral ventricles decreases stress induced food intake and body weight gain (Patterson et al., 2013).

In order to produce these effects, ghrelin may be targeting a number of central sites that contain the ghrelin receptor and that are involved in food intake and metabolic rate, as well as in the regulation of the stress response including the hypothalamic PVN (Guan et al., 1997; Zigman et al., 2006). The present study was designed to examine the contribution of ghrelin receptors in the hypothalamic PVN in modulating some of the metabolic effects caused by chronic social defeat stress in mice. The results from the current experiment indicate that chronic ghrelin receptor blockade in the PVN via infusions of [D-Lys-3]-GHRP-6, does not attenuate stress induced increases in caloric intake or body weight, nor does it affect plasma levels of glucose. Interestingly, while stress decreased the intake of a high fat diet that was provided for 4 h during the day, [D-Lys-3]-GHRP-6 treated mice tended to consume more of this diet compared to animals receiving vehicle infusions and this effect was independent from stress. Not surprisingly, [D-Lys-3]-GHRP-6 treated mice weighed more than vehicle treated mice. Furthermore, these animals also tended to have elevated levels of circulating CORT regardless of the treatment. These effects were not observed in the few animals that received [D-Lys-3]-GHRP-6 infusions into areas outside the PVN (i.e., missed cannula placements) supporting the evidence that GHSR signaling in the PVN selectively mediates these effects.

Given that acute direct infusion of ghrelin into the PVN increase food intake in rats (Currie et al., 2005) we expected that stress induced increases in caloric intake would be attenuated by chronic blockade of ghrelin receptors using [D-Lys-3]-GHRP-6. Nevertheless, [D-Lys-3]-GHRP-6 was ineffective in decreasing caloric intake. In contrast, work from our lab has demonstrated that [D-Lys-3]-GHRP-6 delivered into the ventricles while animals are being stressed does decrease stress induced caloric intake (Patterson et al., 2013). It is therefore likely that, when infused into the ventricles, [D-Lys-3]-GHRP-6 blocks the ghrelin receptor in regions of the brain other than the PVN to reduce feeding including the ventromedial hypothalamus (VMH), arcuate nucleus (ARC), ventral tegmental area (VTA) and brain stem nuclei like the parabrachial nucleus and nucleus of the solitary tract (NTS), all implicated in the feeding responses to ghrelin (Tschop et al., 2000; Nakazato et al., 2001; Faulconbridge et al., 2003; Naleid et al., 2005; Abizaid et al., 2006; Skibicka et al., 2011). In this sense, central blockade of ghrelin receptors may attenuate the release of agouti related peptide (AgRP) and neuropeptide Y (NPY) from the ARC, attenuate dopaminergic tone from the VTA and noradrenergic tone from the brain stem to decrease food intake all resulting in overall attenuated feeding responses to chronic social stress. In our study, however, chronic blockade of the ghrelin receptor in the PVN was not sufficient to attenuate overall caloric intake.

In contrast, we did observe an increase in the intake of the palatable diet in those animals that received [D-Lys-3]-GHRP-6 into the PVN regardless of whether they were stressed or not. This would suggest that ghrelin receptors in the PVN, while not necessary for the overall feeding response seen during chronic social defeat, may be necessary for the switch in dietary preference that has been observed in stressed animals in previous studies (Patterson et al., 2013). In these studies, mice with restricted access to a high fat diet to 4 h during the day gradually increase the proportion of calories consumed from this high fat diet while decreasing the proportion of calories consumed from a regular diet, indicating a bias toward the calorically dense and highly palatable diet. When stressed, however, these mice increased the intake of regular chow while decreasing the intake of the high fat diet (Patterson et al., 2013). It is therefore, possible that stress induced ghrelin secretion acts on the PVN to inhibit the intake of high fat without altering total caloric intake. This would be in accordance with data showing that the PVN is important for dietary preference for carbohydrates and lesions to the PVN result in increased preference for high fat diets (Aravich and Sclafani, 1983; Leibowitz, 1988; Hoebel et al., 1989). Interestingly, ghrelin decreases inhibitory post synaptic currents in parvocellular neurons within the PVN, primarily cells that secrete CRH (although also in some TRH and other cells of unknown phenotype), suggesting that ghrelin facilitates indirect activation of these neuroendocrine cell groups (Cowley et al., 2003). Furthermore, central ghrelin infusions increase extracellular norepinephrine content in the PVN (Kawakami et al., 2008). Of course these effects are acute and it is not known if they are the same in animals subjected to chronic stress. Thus, while plasma ghrelin and CORT levels correlate during acute stress, ghrelin secretion remains elevated during chronic stress but the CORT response become blunted (Zheng et al., 2009). Interestingly, our data shows that chronic GHSR antagonism in the PVN results in higher plasma CORT levels, an effect that seems more pronounced in stressed animals (although not statistically significant). This would suggest that ghrelin may be important in the adaptive mechanisms that bring CORT levels down in the face of chronic stress. As such, one would expect that chronic blockade of GHSR in the PVN would make mice more vulnerable to stress induced pathological conditions, a state previously shown in GHSR KO mice (Lutter et al., 2008).

Finally, it is important to note that in our studies cannulae placements were closer to the magnocellular portion of the PVN, where cells that produce oxytocin, vasopressin, and galanin are located (Akabayashi et al., 1994). All of these cell groups have been associated with feeding, and ghrelin may influence the expression of these peptides in the PVN directly or indirectly (Akabayashi et al., 1994; Sclafani et al., 2007; Beck and Max, 2008). Thus, one could argue that stress induced ghrelin secretion targets the PVN to alter the preference of high fat diets via modulation of oxytocin, vasopressin and galanin, a hypothesis that needs further study.

Possible limitations of this study are the use of [D-Lys-3]-GHRP-6 to antagonize the ghrelin receptor. For instance, [D-Lys-3]-GHRP-6 appears to interact with the 5-HT_2b_ receptors in the gut (Depoortere et al., 2006) although this interaction needs to be further demonstrated, as brain expression of this receptor is low compared to the gut (Hayes and Greenshaw, 2011). Given this finding, and the fact that other ghrelin receptor antagonists appear to act centrally to cause weight gain in spite of blocking the somatotropic effects of ghrelin (Halem et al., 2005), support the notion that the effects of [D-Lys-3]-GHRP-6, are related to their interaction with the ghrelin receptor and not to other receptors.

In summary, our results further demonstrate that chronic social defeat stress results in increased caloric intake and preference for carbohydrate rich diets in mice. This preference for carbohydrate (but not the overall increase in caloric intake) is mediated by direct action of stress induced ghrelin secretion on the PVN. Blockade of GHSR in the PVN biases animals toward increasing their intake of high fat, whether they are stressed or not. As such, the stress induced increases in caloric intake seen in vehicle treated mice, and the increases in the intake of the high fat diet are independent from each other and only the preference for fat is related to blockade of ghrelin receptors in the PVN.

### Conflict of interest statement

The authors declare that the research was conducted in the absence of any commercial or financial relationships that could be construed as a potential conflict of interest.
